# P-591. Injecting Hope: A Long-Acting Injectable Program to Treat HIV Using Cabotegravir/Rilpivirine at a Ryan-White Funded HIV Clinic

**DOI:** 10.1093/ofid/ofae631.789

**Published:** 2025-01-29

**Authors:** Victoria Vega, Kelsea Aragon, Sara Azimi, Jeremy Snyder, Michelle Iandiorio, Bernadette Jakeman

**Affiliations:** University of New Mexico College of Pharmacy, Bosque Farms, New Mexico; University of New Mexico College of Pharmacy, University of New Mexico Medical Group Truman Health Services, Albuquerque, New Mexico; University of New Mexico Hospitals, Albuquerque, New Mexico; University of New Mexico Medical Group, Truman Health Services, Albuquerque, New Mexico; University of New Mexico Medical Group, Truman Health Services, Albuquerque, New Mexico; University of New Mexico, Albuquerque, New Mexico

## Abstract

**Background:**

Cabotegravir/rilpivirine (CAB/RPV) was approved as the first long-acting injectable (LAI) antiretroviral (ART) switch regimen. While clinical trials demonstrate CAB/RPV safety and efficacy, access and clinical workflow challenges have hindered implementation in practice. The objective of this study was to describe use of CAB/RPV in a Ryan-White funded clinic.

**Methods:**

A retrospective chart review was conducted on the first 40 patients, aged > 18 years, referred to the pharmacist CAB/RPV clinic. Referral criteria included HIV-1 RNA < 50 copies/mL and no baseline CAB/RPV resistance.

**Results:**

The majority of patients were male (90%) and white (85%), with a median age of 48 years (range 20-79), BMI of 28 (range 21-48), and use of 6 non-HIV medications (range 0-25). 73% had prior non-adherence to appointments. A DNA archive genotype was obtained for 8 patients due to lack of data. 4 patients had baseline RPV mutations, 2 were identified post-DNA archive genotyping. 14 patients did not start CAB/RPV (6 opted against, 4 had resistance, 2 had insurance denial, 1 had a high copay, 1 moved). The age of those who opted against LAI was not different from those who switched (p=0.2). Pharmacists devoted significant time to patient evaluation and coordination. 26 patients started CAB/RPV; 50% covered by medical benefit, 46% aged > 50 years. Psychological stress/stigma of daily ART was the main reason for switching. 1 patient travelled 228 miles for injections and 1 was homeless. By month 12, 24 patients (92%), including 1 unhoused patient, maintained control on CAB/RPV. No HBV reactivations occurred. 1 patient discontinued due to soreness. 1 patient (BMI < 30, no baseline resistance) had ongoing viremia of < 200 and was switched back to oral ART; post-CAB/RPV DNA archive genotype revealed no mutations.

**Conclusion:**

CBV/RPV offers a switch option for patients, even those with unstable housing or history of missed appointments. Older patients on multiple medications still sought LAI CAB/RPV. Our experience shows that DNA archive genotype testing was valuable in guiding treatment decisions for patients lacking prior data and that proper evaluation and coordination identifies appropriate patients for CAB/RPV, resulting in high continuation rates

**Disclosures:**

**All Authors**: No reported disclosures

